# Lipase-Catalyzed Synthesis, Antioxidant Activity, Antimicrobial Properties and Molecular Docking Studies of Butyl Dihydrocaffeate

**DOI:** 10.3390/molecules27155024

**Published:** 2022-08-07

**Authors:** Bartłomiej Zieniuk, Chimaobi James Ononamadu, Karina Jasińska, Katarzyna Wierzchowska, Agata Fabiszewska

**Affiliations:** 1 Department of Chemistry, Institute of Food Sciences, Warsaw University of Life Sciences—SGGW, 02776 Warsaw, Poland; 2 Department of Biochemistry and Forensic Science, Nigeria Police Academy, Wudil P.O. Box 14830, Nigeria; 3 Department of Food Engineering and Process Management, Warsaw University of Life Sciences—SGGW, 02776 Warsaw, Poland

**Keywords:** antifungal activity, butyl dihydrocaffeate, lipase-catalyzed synthesis, lipophilization, molecular docking, *Rhizopus oryzae*

## Abstract

Green chemistry approaches, such as lipase-catalyzed esterification, are promising methods for obtaining valuable chemical compounds. In the case of the use of lipases, unlike in aqueous environments, the processes of the ester bond formations are encountered in organic solvents. The aim of the current research was to carry out the lipase-catalyzed synthesis of an ester of dihydrocaffeic acid. The synthesized compound was then evaluated for antioxidant and antimicrobial activities. However, the vast majority of its antioxidant activity was retained, which was demonstrated by means of DPPH· (2,2-diphenyl-1-picrylhydrazyl) and CUPRAC (cupric ion reducing antioxidant capacity) methods. Regarding its antimicrobial properties, the antifungal activity against *Rhizopus oryzae* is worth mentioning. The minimum inhibitory and fungicidal concentrations were 1 and 2 mM, respectively. The high antifungal activity prompted the use of molecular docking studies to verify potential protein targets for butyl ester of dihydrocaffeic ester. In the case of one fungal protein, namely 14-α sterol demethylase B, it was observed that the ester had comparable binding energy to the triazole medication, isavuconazole, but the interacted amino acid residues were different.

## 1. Introduction

**Figure 1:**
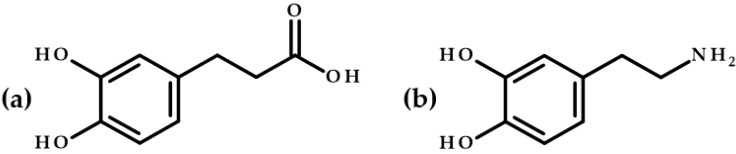
Chemical structures of (**a**) dihydrocaffeic acid and (**b**) dopamine.

Dihydrocaffeic acid (3-(3,4-dihydroxyphenyl)propanoic acid, DHCA) molecule resembles a dopamine (2-(3,4-dihydroxyphenyl)ethylamine) molecule structure and consists of a catechol moiety (a benzene ring with two hydroxyl groups located relative to each other in the *ortho*-position) and a three-carbon side chain with the carboxyl functional group (Figure 1). It can be isolated from such plants as *Gynura bicolor*, *Nepeta teydea*, *Selaginella stautoniana*, and is also present in black olive pericarp [1,2]. Dihydrocaffeic acid is one of the major metabolites of chlorogenic and caffeic acids formed by intestinal bacteria [3]. DHCA was found in blood and urine after consuming the following products: coffee, artichoke leaf extracts, chocolate, or red wine [2]. It has been observed in urine even 48 h after coffee ingestion [4].

Furthermore, the described compound is known to have antioxidant, anti-inflammatory, and cytoprotective properties, and had the potential to decrease lipid peroxidation in human plasma and erythrocytes or protect keratinocytes irradiated with UV [1]. In addition, other beneficial activities of dihydrocaffeic acid were acknowledged, and they were, e.g., lipid-lowering, arousal, neuroprotective, or anti-Alzheimer’s effects [5].

Despite so many aforementioned biological activities of DHCA and other phenolic compounds, these substances also have some disadvantages. Unfortunately, phenolic acids suffer from low solubility in organic solvents, hence their application in lipid-based products is limited. In order to increase the bioavailability, solubility, and stability of phenolic compounds, Esfanjani et al. [6] described the possibility of using innovative nanoencapsulation technologies using lipid substances as carriers, enabling the appropriate and targeted production of functional food. Another way to change the solubility and biological properties of chemical compounds is their lipophilization using biotechnological methods, such as biocatalysis and biotransformation [7]. Lipophilization can be understood by increasing the solubility of a chemical substance in the organic medium and thus in lipids via its structure modification. The basic enzymatic method of modification of phenolic compounds is their esterification with the use of lipases. In many cases, the esterification of chemical compounds apart from improving their solubility in organic environments ameliorates their antioxidant and antimicrobial properties [8,9].

The current work was aimed at the synthesis of an ester of dihydrocaffeic acid via lipase-catalyzed esterification with 1-butanol. The obtained compound as intended was supposed to be more lipophilic compared to the phenolic precursor. Ester’s antioxidant activity was determined by means of DPPH· (2,2-diphenyl-1-picrylhydrazyl) and CUPRAC (cupric ion reducing antioxidant capacity) methods. To assess its modified hydrophilic–lipophilic balance, the ester was set together with known antioxidants in different environments. The antimicrobial properties of butyl dihydrocaffeate and its precursors, i.e., dihydrocaffeic acid and 1-butanol were also evaluated against six bacteria (three Gram-positive and three Gram-negative strains), as well as one filamentous fungi strain. Additionally, to our best knowledge, butyl dihydrocaffeate was for the first time under consideration as a mucormycosis agent with the use of molecular docking studies.

## 2. Results and Discussion

### 2.1. Enzymatic Synthesis and Evaluation of Antioxidant Activity of Butyl Dihydrocaffeate

**Figure 2:**
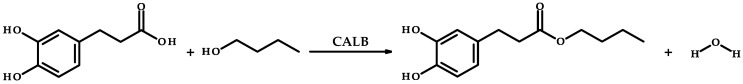
Synthesis of dihydrocaffeic acid butyl ester catalyzed by lipase B from *C. antarctica* (CALB).

The use of lipase from *C. antarctica* in the reaction between dihydrocaffeic acid and 1-butanol (Figure 2) made it possible to obtain the butyl ester of this acid. The mentioned ester (BDHC) was successfully synthesized in order to increase the lipophilicity of phenolic acid. The yield of enzymatic synthesis of dihydrocaffeic acid butyl ester after 72 h was approximately 67%.

Interestingly, biocatalysis, i.e., the use of enzymes in the synthesis, is preferred over conventional chemical catalysis. Enzymes can act in mild conditions, but they are still active in high temperatures and in organic solvents. One of the twelve principles of green chemistry indicates the preference for catalytic reactions, and moreover, enzymes are biodegradable and in comparison with chemical catalysts cause lower energy consumption, less pollution, and produce fewer by-products [7,9].

The functional properties of dihydrocaffeic acid are well-known in the scientific literature, but its products of esterification reactions were researched with slightly less interest, and mainly the antioxidant properties were examined [10,11]. The possibility of designing compounds with high biological activity as well as having good solubility in various environments, including those with high lipid content, is necessary to ensure safe food. Free radicals that appear in food can pose a serious problem to human and animal health. For a more meaningful comparison butyl ester of dihydrocaffeic ester was set with other well-known antioxidants, i.e., butylated hydroxytoluene (BHT), and dihydrocaffeic, L-ascorbic, gallic or caffeic acids in the DPPH· and CUPRAC methods. In the case of the former method, three different solvents were used, i.e., methanol, ethyl acetate, and chloroform to compare also the polarity of solvents and their impact on the antioxidant capacity of tested compounds. The results in the form of the IC_50_ values (the concentration required for a 50% reduction of the DPPH· radical) were summarized in Table 1.

Enzymatically obtained ester was relatively quite stable in its antioxidant activity when compared to different environments. The IC_50_ values presented herein ranged from 0.16–0.22 mM, and these values increased with decreasing the polarity of the applied solvents. Thus, the more non-polar the environment, the weaker the activity of BDHC against the DPPH radical was. Amongst tested compounds, the highest antioxidant properties were exhibited independently of the used solvents by phenolic acids, namely gallic (0.09–0.17 mM), dihydrocaffeic (0.12–0.15 mM), and caffeic (0.13–0.17 mM) acids.

In methanol, which is routinely used solvent in the DPPH· method, ascorbic acid showed lower activity (0.28 mM), and the lowest activity with the IC_50_ value of 0.58 mM was shown by BHT, which in its structure contains only one hydroxyl group. In the case of the rest phenolic compounds tested, the antioxidant activity was decreased in the following order: gallic acid ≈ dihydrocaffeic acid > caffeic acid > butyl dihydrocaffeate. Similarly to the other works, the trend that the number of hydroxyl groups in the aromatic ring has a decisive influence on the activity was maintained [12,13].

In the case of a change in polarity towards more non-polar solvents, i.e., ethyl acetate and chloroform, slight changes in activity were observed for the phenolic acids and the synthesized ester. When ethyl acetate was used as the main solvent in this method, the radical scavenging activities were slightly weaker, and no statistical difference was observed within gallic, caffeic, and its unsaturated derivative acids and the IC_50_ values were 0.13–0.14 mM. The results are interesting for ascorbic acid and BHT, for which it is clearly visible that the applied solvent influenced the final result of the antioxidant activity. The three previously mentioned phenolic acids, with the use of another solvent, i.e., chloroform again proved to be the best in scavenging the DPPH radical. The IC_50_ values ranged from 0.15 to 0.17 mM, followed by butyl dihydrocaffeate with the value of 0.22 mM, and the lowest values were observed for L-ascorbic acid (3.25 mM) and BHT (16.60 mM).

The DPPH· assay is one of the most frequently used methods of assessing antioxidant activity, due to, e.g., the stability of the radical used and its commercial availability, non-specificity, and the ease of implementation of the method [14]. Experiments with the use of this radical have been performed for many years under different conditions, hence the comparison of the results between various research is not the easiest one. Pyrzynska and Pękal [14] found that the results of antioxidant capacity may be influenced by organic solvents, pH, the addition of water, or the presence of metal ions. The study of Dawidowicz et al. [15] showed that the type, as well as the amount of the solvent used in the DPPH· method significantly affected the antioxidant activity of BHT. According to their results, ethyl acetate and dioxane decreased the kinetics of the performed reaction in comparison with the use of methanol. In the case of chloroform, the amount of it was crucial for the influence on the antioxidant properties. It proved that the small amounts of chloroform accelerated the DPPH·/BHT reaction kinetics, but the larger volumes reduced the reaction rate.

Wołosiak et al. [16] evaluated the applicability of ABTS·+ (2,2′-azino-bis-3-ethylbenzothiazoline-6-sulfonic acid) and DPPH· methods to assess the activity of 14 various antioxidants (i.a. phenolic acids, flavonoids, L-ascorbic acid, amino acids, α-tocopherol, their analogs, and derivatives) and mixtures of these compounds. The authors acknowledged that the reaction environment had a greater influence on the obtained results. Interestingly, the ABTS method proved to be more suitable for amine compounds and ascorbic acid. On the other hand, the DPPH assay is more applicable for phenolic compounds and other compounds of limited polarity, and the use of an appropriate solvent should be matched to the polarity of the antioxidant [16].

In the current study, the CUPRAC method was used as a second assay to compare the antioxidant properties of butyl dihydrocaffeate and other compounds. According to Çelik et al. [17], this method can be successfully applied for the evaluation of the antioxidant capacity of both hydrophilic and lipophilic antioxidants in polar and nonpolar solvent media. The highest activities in the form of the TEAC values were obtained for gallic acid (3.37 ± 0.06), caffeic acid (3.35 ± 0.09), and surprisingly butyl dihydrocaffeate (3.50 ± 0.03). The value achieved for the ester is definitely higher compared to its precursor, where the TEAC value of 2.73 ± 0.08 was obtained for dihydrocaffeic acid. The results presented herein were in opposition to the previous work. As a consequence of enzymatic esterification, vanillyl hexanoate was synthesized and compared with its precursor, vanillyl alcohol in DPPH and CUPRAC tests. In both assays, the more lipophilic compound had lower activity [18]. The same may be concluded after analyzing the data from papers of Roleira et al. [19] and Gaspar et al. [20] where it was stated that phenolic acids exhibited higher antioxidant capacity than their esters or amides, which can be related to the steric hindrances of alkyl groups, but on the other hand, the change of the lipophilicity through esterification enhances the applicability of such derivatives in other systems, e.g., lipid-rich matrices.

The difference in activity between caffeic and dihydrocaffeic acids is also surprising since the structures of these two acids only differ in one double bond in the carbon chain. According to Załuski et al. [21], the presence of the double bond in the carbon chain of hydroxycinnamic acids is an important part of the structure along with a phenolic ring affecting the antioxidant activity. It was found that the double bond near the phenolic ring also plays a role in stabilizing the radical by resonance by the interaction of the π electrons of the ring with the π bond of the side chain; hence, caffeic acid should be a better antioxidant than its saturated derivative, i.e., dihydrocaffeic acid [21].

### 2.2. Antimicrobial Properties of Butyl Dihydrocaffeate

In the current research, the antimicrobial properties of butyl dihydrocaffeate were also assessed. Table 2 presents the results of antimicrobial activity of the obtained ester and its precursors, namely dihydrocaffeic acid and 1-butanol. Minimum inhibitory concentrations (MIC) and minimum microbicidal concentrations (MMC) were determined against seven microorganisms (three Gram-negative bacteria, three Gram-positive bacteria, and one species of fungi). According to the results in Table 2, 1-butanol did not exhibit any activity against tested microorganisms. It can be also observed that *E. coli* PCM 2057 was the most resistant to the action of used compounds. Comparing phenolic compounds, it is not possible to indicate a more active compound, so the influence of lipophilization on the antibacterial activity depends on the tested bacteria. For BDHC MICs against bacteria ranged from 4–16 mM, and MMCs were 8–32 mM. In the case of the acid, these ranges were 2–16 and 4–>64 mM, respectively.

A definite difference was observed in the case of *R. oryzae* DSM 2199 mold. Butyl dihydrocaffeate proved to be the most active compound against this fungi, and the MIC value was 1 mM, and MMC was 2 mM, which means that such concentration resulted in the death of almost all introduced inoculum. In comparison for dihydrocaffeic acid, 32 and >64 mM of MIC and MMC were determined, respectively.

**Figure 3:**
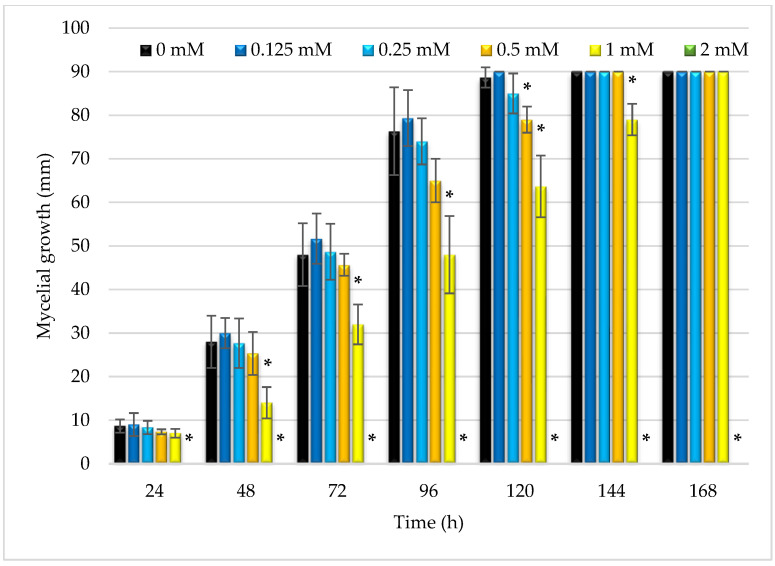
Comparison of the diameter of the *R. oryzae* DSM 2199 mycelium on PDA medium containing the tested ester in concentrations of 0–2 mM. Asterisks (*) annotate the statistical difference (by Dunnett test) in inhibiting the mycelial growth by the tested compound in selected concentration in comparison with control (0 mM). For 2 mM ester concentration no *R. oryzae* growth was observed.

**Figure 4:**
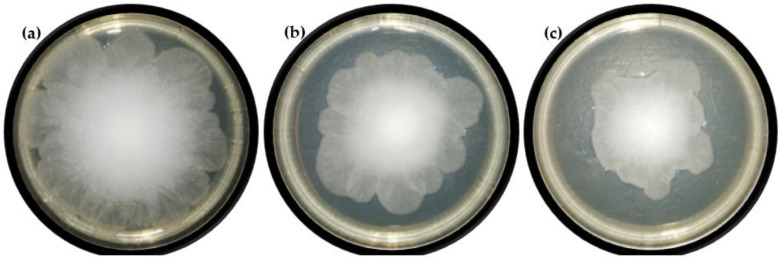
Photographs of *R. oryzae* mycelium after 4 days of cultivation on PDA medium containing the tested ester at the concentration of (**a**) 0, (**b**) 0.5 and (**c**) 1 mM, respectively.

Due to the interesting activity of the obtained ester against fungi, it was decided to evaluate the effect of this substance on the growth of mycelium in a test carried out on agar plates. In six tested concentrations (0–2 mM) over seven days the diameters of the *R. oryzae* DSM 2199 mycelia on the PDA medium were determined. As can be seen in Figure 3, the highest tested concentration of butyl ester, i.e., 2 mM, since the first day of analysis completely inhibited the growth of the fungi. It was confirmed that the concentration used was the minimum fungicidal concentration. Two- and four-times lower concentrations of the tested ester, namely 1 mM and 0.5 mM (Figure 4) were also able to inhibit the mycelium growth, admittedly much weaker than the concentration of 2 mM, but still this change was statistically significant and was confirmed by Dunnett test.

To the authors’ best knowledge, butyl dihydrocaffeate and other lipophilized ester derivatives of dihydrocaffeic acid were never assessed as antibacterial or antifungal agents. This makes it difficult to assess the effect of the alkyl chain elongation on antimicrobial activity. Based on the results of other researchers dealing with different phenolic acids and their enzymatic modification, the positive influence of lipophilization of phenolics on antimicrobial activity may be acknowledged.

An example confirming the previous theses may be a lipophilization of ferulic acid with alcohols from four to twelve carbon atoms carried out by the team of Shi et al. [22,23]. The antimicrobial properties of the obtained esters increased with increasing the length of the alkyl chain, and hexyl ferulate proved to be the ester with the highest activity against *E. coli* and *L. monocytogenes* [22,23]. The antibacterial properties of alkyl gallates were also evaluated. Shi et al. [24] revealed that incorporation of the alkyl chain into gallic acid molecules enhanced antibacterial activities. Moreover, octyl gallate which was incorporated into chitosan film was efficient in the preservation of icefish against *E. coli*. Similarly, in the case of modification of gallic acid, octyl gallate had the best antifungal activity against white-rot fungi, i.e., *Lenzites betulina* and *Trametes versicolor* [25].

Lipophilic catechols, such as esters of dihydrocaffeic acid and hydroxytyrosol can be successfully synthesized using tyrosinase. According to Bozzini et al. [26], esters syntheses were carried out in two steps and entirely with the use of enzymes. Tyrosol or 4-hydroxyphenylpropanoic acid were firstly esterified with carboxylic acids/alcohols of various lengths (C2–C4) via *C. antarctica* lipase B. The resulting esters were then oxidized to catechol derivatives using tyrosinase of mushroom origin (*Agaricus bisporus*). In the aforementioned study, hydroxytyrosol esters were obtained with high yields and compared to conventional chemical synthesis, laborious processes of protecting and deprotecting functional groups were avoided. Propanoic and butanoic esters of hydroxytyrosol were the most active compounds against the influenza A virus, and the antiviral activity of catechol compounds were linked with high antioxidant capacity and the presence of lipophilic alkyl chain [26]. Furthermore, ethyl and butyl dihydrocaffeates were able to inhibit herpes simplex virus type 1 (HSV-1) and type 2 (HSV-2), Coxsackie virus type B3 (Cox B3), and Cytomegalovirus (CMV) [27].

### 2.3. Molecular Docking Studies of Butyl Dihydrocaffeate

Candidiasis, aspergillosis, and mucormycosis are considered the most common invasive fungal diseases causing morbidity and mortality [28]. In the Mucorales order, *Rhizopus* species are responsible for more than 70% of mucormycosis cases with 0.005 to 1.7 cases per million people [29]. Immunocompromised individuals are especially at risk of mucormycosis infection, and the main risk factors increasing the occurrence of mucormycosis are corticosteroid therapy, hematologic malignancies, diabetic ketoacidosis, organ transplantation, or burns [28]. Moreover, SARS-CoV-2 which causes COVID-19, in a combination with mucormycosis proved to be fatal and yielded a significant number of deaths [29]. The above-mentioned fungal disease mainly occurs in three variants: rhinocerebral, pulmonary, and cutaneous, but also gastrointestinal, disseminated, and other rare forms [28,29].

*Rhizopus oryzae* is one of the most economically important members of the Mucorales order and is a fungus used in Asian culture, e.g., involved in tempeh production. Moreover, this fungus is considered GRAS (generally recognized as safe) and can be used for human consumption in the U.S. It is also known for the biosynthesis of a large number of hydrolytic enzymes, such as amylases, proteases, or lipases, and produces other valuable metabolites, namely chitin and chitosan, or fumaric and lactic acids [30,31]. Despite such a valuable contribution to the production of metabolites or participation in the fermentation of food products, *R. oryzae* very often may cause the aforementioned disease known as mucormycosis [28,32].

Due to the fact that butyl dihydrocaffeate had a very high growth inhibitory capacity of *R. oryzae* in in vitro tests, it was decided to evaluate the possibility of using this substance as an antifungal compound and to try to find out the probable mechanism of inhibiting the growth of this fungus using molecular docking. The possible applicability of the synthesized ester as an antifungal agent should be performed, and thanks to computational methods selected physicochemical descriptors, pharmacokinetic properties, drug-likeness, and ADME (absorption, distribution, metabolism, and excretion) parameters could be evaluated. Such parameters were summarized and compared between dihydrocaffeic acid and its butyl ester in Table 3.

As can be seen in Table 3 both compounds did not violate Lipinski’s [33] and Veber’s [34] guidelines for the drug-likeness. The numbers of hydrogen bond donors and acceptors were less than the maximal reference value, and in the case of topological polar surface area (TPSA), the obtained values were less than 140 Å². Both phenolics were also characterized by high gastrointestinal absorption properties. A particularly distinguishing feature of the compared compounds is the possibility of crossing the blood–brain barrier, and according to Roleira et al. [19], compounds with LogP values between 1 and 3 have appropriate lipophilicity to cross membranes, especially the blood–brain barrier. The calculated values of LogP for BDHC and DHCA were 2.44 and 0.63, respectively.

**Figure 5:**
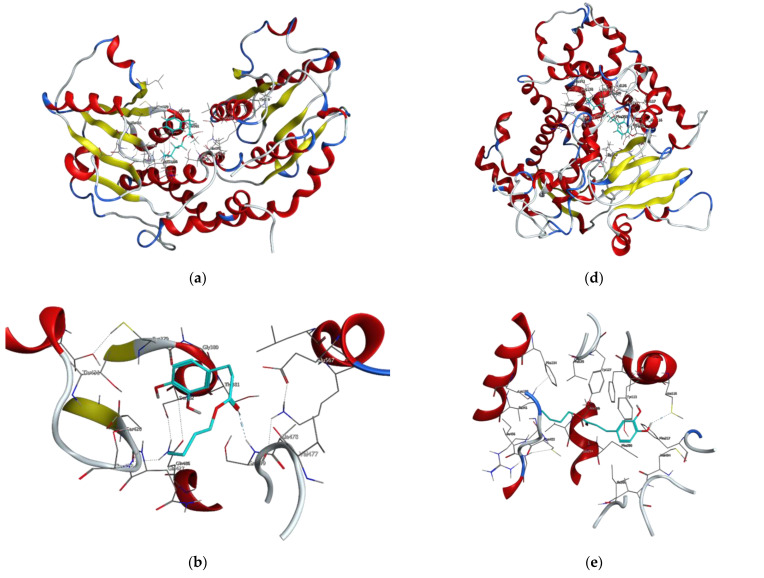

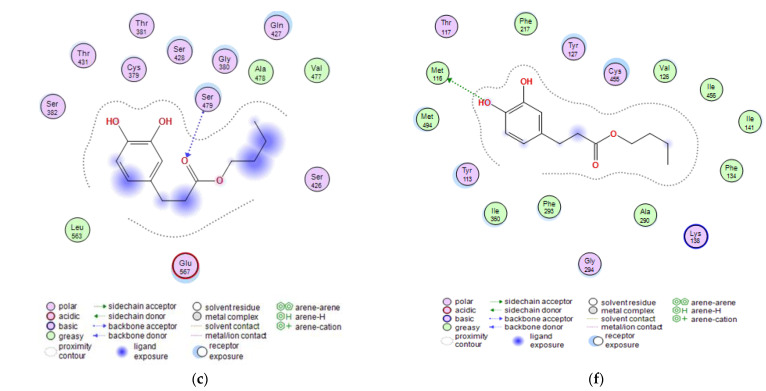
Visualizations of docking analysis of glutamine-fructose-6-phosphate transaminase (GFAT) binding with butyl dihydrocaffeate: (**a**,**b**) 3D visualizations, (**c**) 2D binding interactions, and 14-α sterol demethylase B binding with butyl dihydrocaffeate: (**d**,**e**) 3D visualizations, (**f**) 2D binding interactions.

**Figure 6:**
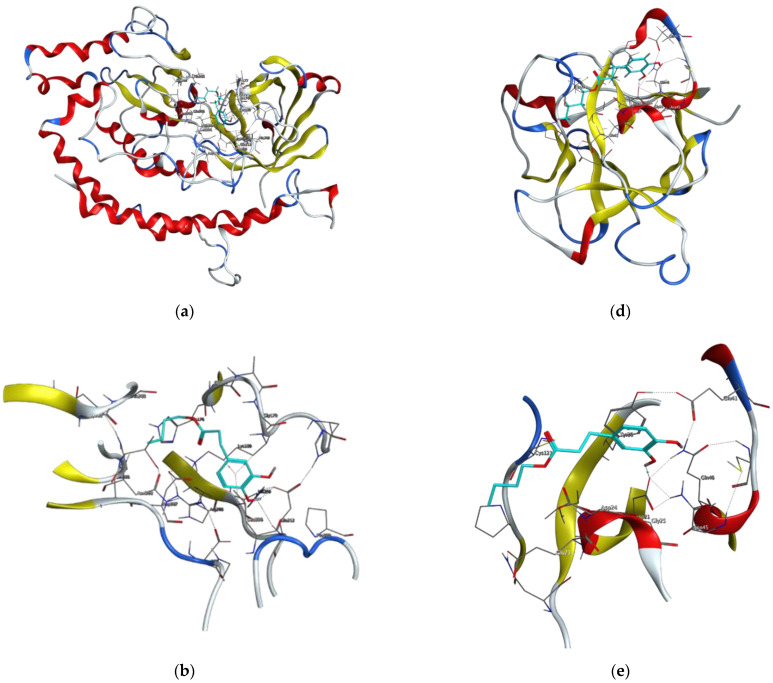

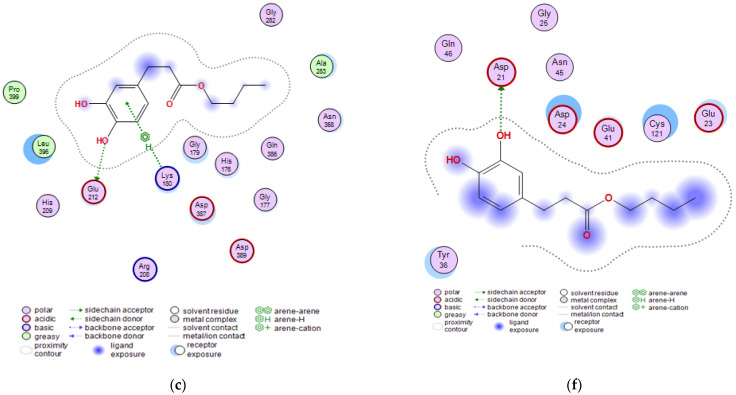
Visualizations of docking analysis of Invasin CotH3 binding with butyl dihydrocaffeate: (**a**,**b**) 3D visualizations, (**c**) 2D binding interactions, and Mucoricin binding with butyl dihydrocaffeate: (**d**,**e**) 3D visualizations, (**f**) 2D binding interactions.

At a later stage of the work, virulence factors of the fungus *R. oryzae* and potential drug targets were searched. Based on the available scientific literature, four proteins have been selected for molecular docking studies. These were the following proteins: glutamine-fructose-6-phosphate transaminase (GFAT), 14-α sterol demethylase B, invasin CotH3, and mucoricin [29,32,35]. Amongst control ligands, posaconazole, isavuconazole, and 12,28-oxamanzamine A were chosen and were compared with dihydrocaffeic acid and its butyl ester, synthesized herein. The results of molecular docking are presented in Figure 5 and Figure 6 and Table 4.

GFAT (EC 2.6.1.16) takes part in the biosynthesis of chitin, a major fungal cell wall component [35]. Control ligands, i.e., triazole antifungals, posaconazole, and isavuconazole, as well as, 12,28-oxamanzamine A, marine-derived macrocyclic alkaloid, had binding energy in the range of −5.8663 to −7.5116 kcal/mol. In the case of DHCA and BDHC, the binding energies were weaker and amounted to −4.1152 and −5.1152, respectively. The binding energy for posaconazole was obtained as −7.5116 kcal/mol, and this antifungal drug interacted with Glu567, Ser428, and Ser382 amino acid residues of GFAT, by making hydrogen bonds or Pi-H bonds. Similarly, the second tested antifungal also interacted with Glu567 and Ser428, and 12,28-oxamanzamine A in addition to Glu567 interacted with Thr381 by hydrogen bonding. Query ligands, namely dihydrocaffeic acid and its butyl ester, due to their similarity in the structures, revealed a common hydrogen bond interaction with Ser479 (Figure 5a–c and Table 4).

Taking into account that the PDB structures of proteins of *R. oryzae* and other Mucorales fungi are not always known, the rapid progress in genome sequencing greatly helped in the search for new antifungal substances. The molecular docking study of Banerjee et al. [35] revealed that peptide inhibitors, specifically N^3^-(4-methoxyfumaroyl)-L-2,3-diaminopropanoic acid and 2-amino-2-deoxy-D-glucitol-6-phosphate may be probable compounds involved in the inhibition of GFAT.

The subsequent protein tested for interaction with the synthesized compound was 14-α sterol demethylase B. This enzyme present in fungi is responsible for the demethylation of lanosterol to an important intermediate, which is then converted into ergosterol, one of the major sterols and the component of fungal cell membranes functionally comparable to cholesterol in animal cells. Due to the fact that fungi cannot survive without ergosterol, the aforementioned enzyme is a target for antifungal drugs, and for this purpose, azole antifungal agents are used [32].

Therefore, posaconazole bound most strongly to the tested protein, and the obtained value was −9.7030 kcal/mol. This triazole antifungal medication interacted with Cys455 by hydrogen bond and with Tyr133 and Phe222 by Pi-H bonds. The second triazole compound had binding energy of −6.1767 kcal/mol and interactions with His453, Gly294, Val291, and Cys455 were observed. In the case of macrocyclic alkaloids, the weakest binding between protein and ligand was noted (−4.0297 kcal/mol), and the ligand was a hydrogen bond donor for Met494. DHCA had a stronger binding affinity compared to 12,28-oxamanzamine A (−4.5334 kcal/mol) and the same type of interaction was observed. Butyl dihydrocaffeate also interacted with methionine (Met116). A hydrogen bond linked the sulfur atom of methionine and a hydroxyl group from the catechol ring of BDHC. The resulting binding energy for such a connection amounted to −6.1416 kcal/mol and was comparable to that of isavuconazole (Figure 5d–f and Table 4).

Prajapati et al. [32] suggested the possibility of the use of another phenolic compound to interact with fungal sterol demethylase. The abovementioned compound, curcumin is the best-known phenolic compound among curcuminoids, being the main ingredient of turmeric (*Curcuma longa*). This compound is associated with a number of biological activities, (e.g., with antifungal activity), and turmeric is a popular ingredient in dietary supplements and used in traditional folk medicine [36]. Computer-aided, but also experimental research was conducted to evaluate the ability of curcumin to interact with sterol demethylase. Through the use of molecular docking, MM-GBSA (Molecular Mechanics with Generalized Born Surface Area), and molecular dynamics simulation the hypothesis was confirmed. Furthermore, in vitro assays acknowledged the antifungal activity of curcumin against *R. oryzae*, and curcumin-dependent inhibition of ergosterol synthesis was observed [32].

The next targeted protein was Invasin CotH3. CotHs are spore coat protein homologs of Mucorales, and they act as fungal ligands for endothelial cell glucose-regulated protein 78 (GRP78), which mediates host cell invasion. According to Gebremariam et al. [37], heterologous expression of CotH2 and CotH3 in *Saccharomyces cerevisiae* admitted the possibility to invade the host cells through binding to GRP78, and CotH proteins can be considered therapeutic target against mucormycosis [37]. Posaconazole, similar to the previously provided results, had the strongest binding energy, and this time it was −8.9723 kcal/mol. The observed interactions were: hydrogen bond with Ala303 and Pi-H bond with Lys180. Isavuconazole and 12,28-oxamanzamine A found to have similar binding energy values, namely −7.3442 and −7.3644, respectively. The first one made a Pi-H bond with Gly179 and Lys180, and the latter with Asn368, Asp387 (ligand act as an H-donor), and His176 (H-Pi interaction), Thr367 (Pi-H bond), and Asp387 (ionic interaction). Dihydrocaffeic acid was a hydrogen bond acceptor for Asn368, and Gln386 and the calculated binding energy was the lowest (−4.9011 kcal/mol). In the case of butyl ester, the binding energy was definitely different than in DHCA and amounted to −6.3490 kcal/mol. The synthesized ester was a hydrogen bond donor for Glu212, and similarly to triazoles, the interaction with Lys180 was noticed (Figure 6a–c and Table 4).

The last protein subjected to molecular docking studies was mucoricin. The proposed name of the protein came from its structural and functional similarities to the plant toxin ricin. It is a 17 kDa toxin, which probably plays a key role in the virulence of Mucorales fungi. This protein due to the N-glycosylase activity has the ability to inhibit protein synthesis. In a similar manner to the above-mentioned proteins, namely GFAT, Invasin CotH3, and sterol demethylase, mucoricin should also be considered a therapeutic target against mucormycosis [38].

Ligands docked to the mucroricin referring to their binding energies can be set in the following order: posaconazole > 12,28-oxamanzamine A > isavuconazole > BDHC > DHCA. The first three ligands interacted with glutamic acid residue through hydrogen bonds, and in the case of posaconazole it was Glu23 and the binding energy was −6.5630 kcal/mol. 12,28-Oxamanzamine A interacted with another glutamic acid residue, i.e., Glu41, and the second triazole antifungal agent in addition to Glu41 had also been in relation with Asp21. Exactly for this amino acid residue (aspartic acid), the interaction was observed for dihydrocaffeic acid and its butyl ester. Despite that the binding energy for butyl ester was stronger than for its precursor (−4.6642 vs. −4.3442), dihydrocaffeic acid interacted also with Lys59, and the acid was the hydrogen acceptor this time (Figure 6d–f and Table 4). It is worth noting that the esterification of dihydrocaffeic acid each time increased the binding energy to selected proteins compared to the acid itself.

Pokharkar et al. [39] have chosen 35 chemical compounds from marine organisms using the PASS online program, and molecular docking and molecular dynamics simulations were performed to assess the possibility of chosen compounds to be a candidate against mucormycosis, and the following protein as potential drug targets were evaluated: CotH3, mucoricin, lanosterol 14α demethylase, exo-1,3-beta-glucan synthase, Rhizopuspepsin, RdRp (RNA-dependent RNA polymerase), and fungal lipase. According to the results, (+)-curcudiol and (+)-curcuphenol, i.e., phenolic derivatives, proved to be the most promising compounds, which exhibited the widest spectrum of inhibition potential [39].

Similarly to the above-cited paper and to the current study, Madanagopal et al. [29] also performed molecular docking studies of different ligands against CotH3, lanosterol 14-α demethylase, and mucoricin. Approximately 300 compounds including bioactive compounds, FDA-approved/unapproved drugs, or investigational-only drugs were applied against these three proteins. Computational studies of the authors allowed the identification of six potential inhibitors of *Rhizopus delemar* proteins, i.e., hesperidin (a flavanone glycoside) for mucoricin; pramiconazole, and saperconazole (triazole drugs) against lanosterol 14-α demethylase, and vialinin B, deoxytopsentin, and 12,28-oxamanzamine A as inhibitors of CotH3. The last one, also used as a control in the current study, exhibited very high values of binding affinities for all tested proteins [29].

## 3. Materials and Methods

### 3.1. Materials

In the current study, immobilized lipase B from *Candida antarctica* (CALB) purchased from Sigma-Aldrich (Poznań, Poland) was used as a biocatalyst in the biotransformation reaction. Chemicals used in the study were acquired from Sigma-Aldrich and Avantor Performance Materials Poland S.A. (Gliwice, Poland). Moreover, culture media and their components were purchased from BTL Sp. z o. o. (Łódź, Poland).

### 3.2. Microorganisms

The following microorganisms were used: *Escherichia coli* PCM 2057, *Enterobacter cloacae* PCM 2848, *Serratia marcescens* PCM 549, *Bacillus subtilis* PCM 486, *Listeria monocytogenes* PCM 2191, *Staphylococcus aureus* PCM 2054 from the Polish Collection of Microorganisms of the Institute of Immunology and Experimental Therapy, Polish Academy of Sciences (Wrocław, Poland), and *Rhizopus oryzae* DSM 2199 from the German Collection of Microorganisms and Cell Cultures (Deutsche Sammlung von Mikroorganismen und Zellkulturen GmbH, DSMZ, Braunschweig, Germany). Bacterial strains were stored in 20% (*v*/*v*) glycerol solution in nutrient broth at −20 °C. Mold spores were stored in sterile 0.85% NaCl solution at 4 °C.

### 3.3. Enzymatic Synthesis of Butyl Dihydrocaffeate

The synthesis of butyl dihydrocaffeate was carried out according to the reaction scheme in Figure 2. In the conical flask dihydrocaffeic acid and 1-butanol in a ratio of 1:1.5 were added and were dissolved in the mixture of methyl-*tert*-butyl ether and isooctane (2:1, *v*/*v*). Subsequently, after substrates dissolved the CALB as a biocatalyst was added (addition of 15% by weight of substrates). The reaction was carried out at 37 °C at 250 rpm on a rotary shaker for 72 h.

The obtained ester was purified with column chromatography. Silica gel 60 (0.040–0.063 mm; 230–400 mesh) was used as a stationary phase and chloroform: methanol mixture (9:1, *v*/*v*) was applied as a mobile phase. Fractions were collected in separate flasks and then analyzed by TLC. Fractions containing ester were then dried with MgSO_4_ and the mixture of solvents was evaporated. Proton (^1^H NMR) and carbon-13 (^13^C NMR) nuclear magnetic resonance spectroscopic analyses were applied to confirm the structure of the obtained ester. Spectra were recorded on a Bruker AVANCE spectrometer (Bruker, Billerica, MA, USA) using CDCl_3_ as a solvent. Chemical shifts of butyl dihydrocaffeate are reported below in ppm (δ) relative to internal standard—tetramethylsilane (TMS).

^1^H NMR (300 MHz, CDCl_3_): δ 0.92 (3H, t, *J* = 7.3 Hz), 1.25–1.41 (2H, m), 1.52–1.64 (2H, m), 2.58 (2H, t, *J* = 7.6 Hz), 2.84 (2H, t, *J* = 7.6 Hz), 4.07 (2H, t, *J* = 6.7 Hz), 5.28 (1H, s), 5.45 (1H, s), 6.62 (1H, dd, *J* = 8.1, 2.0 Hz), 6.71 (1H, d, *J* = 2.0 Hz), 6.76 (1H, d, *J* = 8.1 Hz).

^13^C NMR (75 MHz, CDCl_3_): δ 13.66 (1C, s), 19.07 (1C, s), 30.31 (1C, s), 30.58 (1C, s), 36.16 (1C, s), 64.61 (1C, s), 115.32 (1C, s), 115.36 (1C, s), 120.55 (1C, s), 133.34 (1C, s), 142.01 (1C, s), 143.57 (1C, s), 173.73 (1C, s).

### 3.4. Evaluation of Antioxidant Activity

#### 3.4.1. The DPPH· Assay

To evaluate the antioxidant activity of obtained ester the DPPH· assay was used according to the protocol of Zanetti et al. [40] with slight modifications. Briefly, 0.004% solutions of DPPH· in methanol, ethyl acetate, or chloroform, and stock solutions (concentration = 10 mM) of butyl dihydrocaffeate, as well as dihydrocaffeic, caffeic, gallic, L-ascorbic acids, and BHT (butylhydroxytoluene) in ethanol were prepared. The solutions of the tested compounds to the DPPH· solution were used in the ratio of 1:9 (*v*/*v*). The antioxidant activities of tested compounds were measured by using a Rayleigh UV-1601 spectrophotometer (BRAIC, Beijing, China) at 517 nm. Based on the obtained results the IC_50_ parameters, i.e., the concentration required for a 50% reduction of the DPPH· radical were calculated.

#### 3.4.2. CUPRAC Method

CUPRAC (cupric ion reducing antioxidant capacity) assay was used as a second method to compare the antioxidant activities of tested substances and was performed based on the methodology of Özyürek et al. [41]. In the abovementioned method, the absorption of the formed complex of neocuproine (2,9-dimethyl-1,10-phenanthroline) and Cu(I) ion is measured spectrophotometrically at 450 nm, where antioxidant compounds serve as electron reductants. The Trolox Equivalent Antioxidant Capacities (TEAC) were determined for the tested compounds based on the absorbance of compounds and Trolox, which was used as a reference standard.

### 3.5. Evaluation of Antimicrobial Activity

#### 3.5.1. Minimum Inhibitory Concentration (MIC) Determination

The MIC values of butyl dihydrocaffeate, and its precursors, i.e., dihydrocaffeic acid and 1-butanol were determined by the microdilution broth method on 96-well plates according to ISO [42] against *E. coli* PCM 2057, *E. cloacae* PCM 2848, *S. marcescens* PCM 549, *B. subtilis* PCM 486, *L. monocytogenes* PCM 2191, *S. aureus* PCM 2054, and *R. oryzae* DSM 2199.

#### 3.5.2. Minimum Microbicidal Concentration (MMC) Determination

After reading the MIC values, i.e., after 24 h for bacteria and 48 h for molds, the minimum microbicidal concentrations (MMC) were determined. From wells where no growth was observed 3 µL of microorganism culture was transferred onto Mueller–Hinton agar (BTL Sp. z o. o., Łódź, Poland) for bacteria or Sabouraud agar (BTL Sp. z o. o., Łódź, Poland) for *R. oryzae*. Afterward, agar plates were incubated at 37 °C for 24 h for bacteria or 28 °C for 48 h for the tested mold species, and the growth of the microorganisms allowed for determining the minimum microbicidal concentrations.

#### 3.5.3. Inhibition of Mycelium Growth of *R. oryzae* by Butyl Dihydrocaffeate

The obtained ester, butyl dihydrocaffeate was also evaluated as an inhibitor of the mycelial growth of *R. oryzae*. For this purpose, one milliliter of an ester solution in ethanol of various concentrations (0, 0.125, 0.25, 0.50, 1, and 2 mM) was transferred onto a Petri plate and 19 mL of PDA (Potato Dextrose Agar; BTL Sp. z o. o., Łódź, Poland) was added and mixed thoroughly. After solidification, 10 µL of 1.2 × 10^6^ CFU/mL of *R. oryzae* DSM 2199 spore suspension was applied on the surface of the plate. Plates were incubated at 28 °C for 7 days, and after every 24 h, the diameters of mycelia were measured.

### 3.6. Structures’ Comparison of Dihydrocaffeic Acid and Its Butyl Ester

Selected physicochemical descriptors, pharmacokinetic properties, drug-likeness, and ADME (absorption, distribution, metabolism, and excretion) parameters of dihydrocaffeic acid and butyl dihydrocaffeate were computed and predicted using SwissADME [43]. The drug-likeness evaluation was made of Lipinski’s [33] and Veber’s [34] guidelines.

### 3.7. Statistical Analysis

Statistical analysis was performed using Statistica 13.3 software (TIBCO Software Inc., Palo Alto, CA, USA). The results were analyzed using a one-way analysis of variance (ANOVA) and Tukey’s post hoc test. The Dunnett test was used to compare the butyl dihydrocaffeate inhibitory potential of mycelium growth. The significance level was α = 0.05.

### 3.8. Target Proteins and Ligands Selection for Molecular Docking Studies

Based on the available scientific literature on the molecular docking analysis of selected bioactive compounds against mucormycosis caused by *Rhizopus oryzae*, the following proteins were selected: glutamine-fructose-6-phosphate transaminase (GFAT), 14-α sterol demethylase B, invasin CotH3, and mucoricin [29,32,35]. Amongst control ligands, posaconazole, isavuconazole, and 12,28-oxamanzamine A were chosen and were compared with dihydrocaffeic acid and its butyl ester, synthesized herein.

### 3.9. Protein Modelling and Structure Quality Assessment

The protein structure modeling for the target proteins was performed using the SWISS-MODEL web server [44]. The SWISS-MODEL web server builds a model based on the target-template alignment using ProMod3 [45]. Coordinates that were conserved between the target and the template were copied from the template to the model. Insertions and deletions were remodeled using a fragment library. Sidechains were then rebuilt. Finally, the geometry of the resulting model was regularized by using a force field. The global and per-residue model qualities were assessed using the QMEAN scoring function [45]. The predicted models were validated using ERRAT and PROCHECK [46]. The structures of the predicted models are presented in Figure S1. while the templates used for each target protein and the estimated target-template alignment quality indices (sequence identity, similarity, coverage, and Global Model Quality Estimation (GMQE) value) are presented in Table S1 and Figure S2.

### 3.10. Protein/Ligand Preparation and Docking Simulation

#### 3.10.1. Ligand Selection and Preparation

The 3D structures of the query ligands and the controls were built in Molecular Operating Environment (MOE; Chemical Computing Group, Montreal, QC, Canada) suite using smiles obtained from PubChem. The ligands were then prepared for docking as follows: protonation at a temperature of 300 K and pH 7.0 and energy minimization, using default parameters—Amber10-EHT force field was used with no periodicity, the constraints were maintained at the rigid water molecule level and partial charges were also applied [47]. Following the preparation process, the ligands were organized in a database for simultaneous virtual screening (docking).

#### 3.10.2. Protein Target Preparation and Binding/Docking Site Prediction

The predicted and validated models of the target proteins were prepared for docking using tools and protocols in MOE also. The preparatory process included the removal of water molecules and other heteroatoms. Protonation, partial charges, and energy minimization were implemented as described above in ligand preparation. The fully prepared and optimized 3D structure was saved in *moe* format for docking [47]. The active site of the predicted model was computed or determined using the *site finder* protocol in MOE [47].

#### 3.10.3. Docking Simulation

The docking simulations were performed in MOE using the Triangular matcher/rigid receptor method and scored using Affinity dG/GBVI/WSA dG options, on an Intel Core i7 CPU @ 2.00 GHz, 2.60 GHz. The validation of the docking program and its scoring function was implemented as described by Ononamadu et al. [47]. Thus, the default methods and scoring function of MOE were adopted for this study. The triangular matcher method (default in MOE) was adjudged the best placement method for standard and well-defined binding sites in MOE [48]. It generated poses by superimposing triplets of ligand atoms and triplets of receptor sites (alpha centers that represent locations of tight packing) [48]. The poses generated by the placement method were scored by the selected scoring function, Affinity dG, and subsequently re-scored by GBVI/WSA dG. The Affinity dG is an empirical scoring function that calculates enthalpic contribution to binding energy using a linear function based on the following interaction factors: hydrogen bond donor−acceptor, pair, ionic interactions, metal ligation, hydrophobic interaction, and interactions between hydrophobic and polar atoms and between any two atoms [49]. The GBVI/WSA dG SF on the other hand is a force field-based scoring function that estimates the binding affinity of the ligand based on Coulombic electrostatic, solvation electrostatic, van der Waals, and surface area terms trained with MMFF94x and AMBER99 force fields and ninety-nine (99) protein−ligand complexes of the solvated interaction energy (SIE) training set [50]. The protein–ligand docking poses and scores were saved in *db* format and ligand interaction with protein was visualized (2D and 3D) using Discovery studio and MOE ligand interaction options [47].

## 4. Conclusions

The current work presents the synthesis of butyl dihydrocaffeate, and the enzymatic process applied herein can be an attractive alternative to traditional chemical synthesis. The obtained compound was characterized by a high antifungal activity against *R. oryzae*, which is one of the causative agents of mucormycosis. As assumed, the resulting ester was more lipophilic than its precursor—dihydrocaffeic acid and the evidence from this study implies that butyl dihydrocaffeate could be a potential food additive with antioxidant and/or antifungal properties. It seems interesting that the obtained ester had comparable binding energy to one fungal protein, namely 14-α sterol demethylase B as isavuconazole (a triazole drug). This indicates a possible mechanism of action of dihydrocaffeic acid ester on *R. oryzae*, but further research is needed. Future studies could investigate also the use of dihydrocaffeic acid derivatives and the impact of the alkyl chain length on the oxidative stability of lipid-rich matrices.

## Figures and Tables

**Table 1:** Comparison of the antioxidant activity of dihydrocaffeic acid, its butyl ester, and commonly known antioxidants by DPPH· and CUPRAC methods.

	DPPH·			CUPRAC
Compound	Methanol (mM)	Ethyl Acetate (mM)	Chloroform (mM)	(TEAC)
BDHC	0.16 ± 0.01 ^Ca^	0.18 ± 0.01 ^Ba^	0.22 ± 0.01 ^Bb^	3.50 ± 0.03 ^A^
DHCA	0.12 ± 0.01 ^Aa^	0.14 ± 0.01 ^Ab^	0.15 ± 0.01 ^Ab^	2.73 ± 0.08 ^B^
LAA	0.28 ± 0.04 ^Da^	0.23 ± 0.02 ^Ca^	3.25 ± 0.08 ^b^	1.56 ± 0.01 ^D^
BHT	0.58 ± 0.03 ^Ea^	21.34 ± 1.55 ^c^	16.60 ± 1.79 ^b^	1.92 ± 0.01 ^C^
GA	0.09 ± 0.01 ^Aa^	0.13 ± 0.1 ^Ab^	0.17 ± 0.01 ^Ac^	3.37 ± 0.06 ^A^
CA	0.14 ± 0.01 ^Ba^	0.13 ± 0.01 ^Aa^	0.17 ± 0.01 ^Ab^	3.35 ± 0.09 ^A^

Abbreviations: BDHC: dihydrocaffeic acid butyl ester, DHCA: dihydrocaffeic acid, LAA: L-ascorbic acid, BHT: butylated hydroxytoluene, GA: gallic acid, CA: caffeic acid, TEAC: Trolox equivalent antioxidant capacity. Values with different capital letters in the column (A–E) differ statistically (α = 0.05). Values with different lowercase letters in the row within DPPH·method (a–c) differ statistically (α = 0.05).

**Table 2:** Comparison of the antimicrobial activity of dihydrocaffeic acid butyl ester and its precursors.

	BDHC		DHCA		1-Butanol	
	MIC (mM)	MMC (mM)	MIC (mM)	MMC (mM)	MIC (mM)	MMC (mM)
*E. coli* PCM 2057	16	32	16	32	32	64
*E. cloacae* PCM 2848	8	16	2	4	16	32
*S. marcescens* PCM 549	4	8	16	32	16	32
*B. subtilis* PCM 486	8	16	2	>64	16	>64
*L. monocytogenes* PCM 2191	8	16	8	16	16	64
*S. aureus* PCM 2054	4	8	2	4	16	>64
*R. oryzae* DSM 2199	1	2	32	>64	32	>64

Abbreviations: BDHC: dihydrocaffeic acid butyl ester, DHCA: dihydrocaffeic acid, MIC: minimum inhibitory concentration, MMC: minimum microbicidal concentration.

**Table 3:** Comparison of selected physicochemical descriptors, pharmacokinetic properties, drug-likeness, and ADME (absorption, distribution, metabolism, and excretion) parameters of dihydrocaffeic acid butyl ester and its precursor.

	DHCA	BDHC	Reference Value *
Molecular weight (g/mol)	182.17	238.28	<500
Hydrogen bond donors	3	2	<5
Hydrogen bond acceptors	4	4	<10
Rotatable bonds	3	7	<10
Molar refractivity	46.48	65.58	
TPSA (Å²)	77.76	66.76	≤140
LogP	0.63	2.44	<5
LogS	−0.62 (Very soluble)	−4.42 (Moderately soluble)	
Rule of five violations	0	0	
Gastrointestinal absorption	High	High	
The blood–brain barrier permeant	No	Yes	

* The drug-likeness reference values declared based on Lipinski’s [33] and Veber’s [34] guidelines. Abbreviations: DHCA: dihydrocaffeic acid, BDHC: dihydrocaffeic acid butyl ester, TPSA: topological polar surface area, LogP: partition coefficient (measure of lipophilicity), LogS: water solubility.

**Table 4:** Molecular docking scores of the query ligands docked to target proteins (glutamine-fructose-6-phosphate transaminase (GFAT), 14-α sterol demethylase B, Invasin CotH3, and Mucoricin).

Target Protein	Compound	Binding Energy (kcal/mol)	Interacting Amino Acid Residues
Glutamine-fructose-6-phosphate transaminase (GFAT)	Posaconazole	−7.5116	^a^ Glu567, ^b^ Ser428, ^c^ Ser382
	Isavuconazole	−5.8663	^a^ Glu567, ^b^ Ser428
	12,28-Oxamanzamine A	−6.1423	^b^ Glu567, ^b^ Thr381
	DHCA	−4.1152	^b^ Ser479
	BDHC	−5.1152	^b^ Ser479
14-α sterol demethylase B	Posaconazole	−9.7030	^a^ Cys455, ^c^ Tyr133, ^c^ Phe222
	Isavuconazole	−6.1767	^a^ His453, ^b^ Gly294, ^c^ Val291, ^c^ Cys455
	12,28-Oxamanzamine A	−4.0297	^a^ Met494
	DHCA	−4.5334	^a^ Met494
	BDHC	−6.1416	^a^ Met116
Invasin CotH3	Posaconazole	−8.9723	^a^ Ala303, ^c^ Lys180
	Isavuconazole	−7.3442	^c^ Gly179, ^c^ Lys180
	12,28-Oxamanzamine A	−7.3644	^a^ Asn368, ^a^ Asp387, ^e^ His176, ^c^ Thr367, ^d^ Asp387
	DHCA	−4.9011	^b^ Asn368, ^b^ Gln386
	BDHC	−6.3490	^a^ Glu212, ^c^ Lys180
Mucoricin	Posaconazole	−6.5630	^a^ Glu23
	Isavuconazole	−5.4709	^a^ Glu41, ^a^ Asp21
	12,28-Oxamanzamine A	−5.5611	^a^Glu41
	DHCA	−4.3442	^a^ Asp21, ^b^ Lys59
	BDHC	−4.6642	^a^ Asp21

^a^: H-donor, ^b^: H-acceptor, ^c^: Pi-H bond, ^d^: Ionic, ^e^: H-Pi, Abbreviations: DHCA: dihydrocaffeic acid, BDHC: dihydrocaffeic acid butyl ester.

## Data Availability

The data presented in this study are available on request from the corresponding author (B.Z.).

## Supplementary Materials

The following supporting information can be downloaded at: https://www.mdpi.com/article/10.3390/molecules27155024/s1, Raw NMR data, (e.g., fid files), ^1^H NMR and ^13^C NMR spectra of the synthesized compound; Figure S1: Structures of the modeled proteins (**a**) glutamine-fructose-6-phosphate transaminase, (**b**) 14-α sterol demethylase B, (**c**) invasin CotH3, (**d**) mucoricin; Figure S2: The Ramachandran’s plots (PROCHECK) of the modeled proteins (**a**) glutamine-fructose-6-phosphate transaminase, (**b**) 14-α sterol demethylase B, (**c**) invasin CotH3, (**d**) mucoricin; Table S1: The estimated target-template alignment/predicted model quality indices.
