# Supplementary material for: Lipase-Catalyzed Synthesis, Antioxidant Activity, Antimicrobial Properties and Molecular Docking Studies of Butyl Dihydrocaffeate
Source: Molecules. 2022 Aug 7;27(15):5024. doi: 10.3390/molecules27155024 (PMC9370587; doi:10.3390/molecules27155024)

Parameter	Value
Comment	42094-13C<br>B1
Origin	Bruker BioSpin GmbH
Solvent	CDCl3
Temperature	298.2
Pulse Sequence	zgpg30
Number of Scans	1333
Receiver Gain	200
Relaxation Delay	1.5000
Pulse Width	10.0000
Acquisition Time	1.7302
Acquisition Date	2022-08-03T13:33:17
Spectrometer Frequency	75.49
Spectral Width	18939.4
Lowest Frequency	-791.2
Nucleus	13C
Acquired Size	32768
Spectral Size	65536

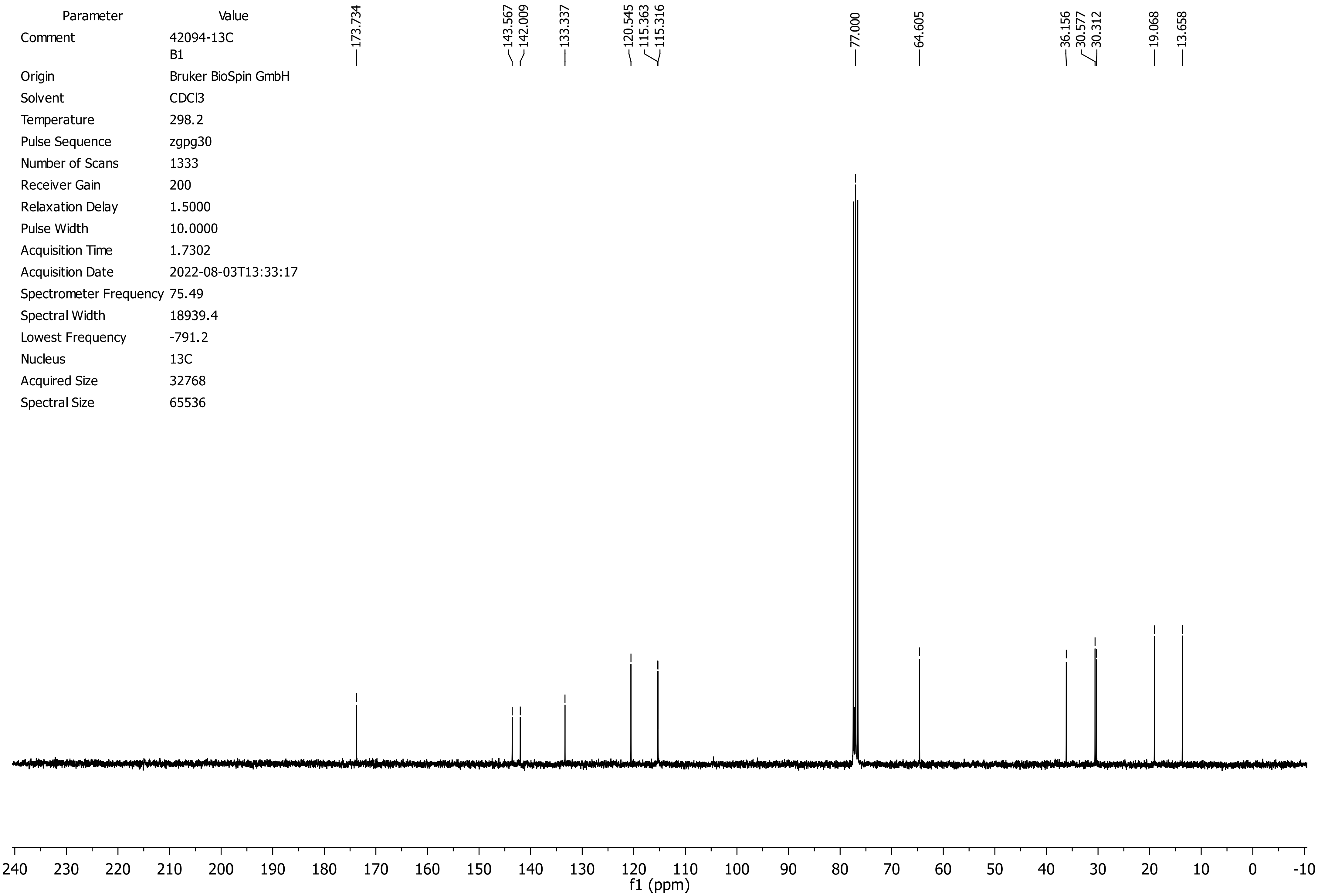

Supplement: Supplementary file 1 [file molecules-27-05024-s001.zip › 13CNMRfiles/1.pdf]
