# Supplementary material for: Lipase-Catalyzed Synthesis, Antioxidant Activity, Antimicrobial Properties and Molecular Docking Studies of Butyl Dihydrocaffeate
Source: Molecules. 2022 Aug 7;27(15):5024. doi: 10.3390/molecules27155024 (PMC9370587; doi:10.3390/molecules27155024)

	Parameter	Value
1	Comment	33270-1H<br>BDHCA
2	Origin	Bruker BioSpin GmbH
3	Owner	nmr
4	Spectrometer	spect
5	Solvent	CDCl3
6	Temperature	298.1
7	Pulse Sequence	zg30
8	Number of Scans	40
9	Receiver Gain	200
10	Relaxation Delay	0.0000
11	Pulse Width	14.5000
12	Acquisition Time	3.5001
13	Acquisition Date	2021-02-11T15:26:52
14	Modification Date	2021-02-11T15:27:53
15	Spectrometer Frequency	300.20
16	Spectral Width	6009.6
17	Lowest Frequency	-1158.2
18	Nucleus	1H
19	Acquired Size	21034
20	Spectral Size	65536

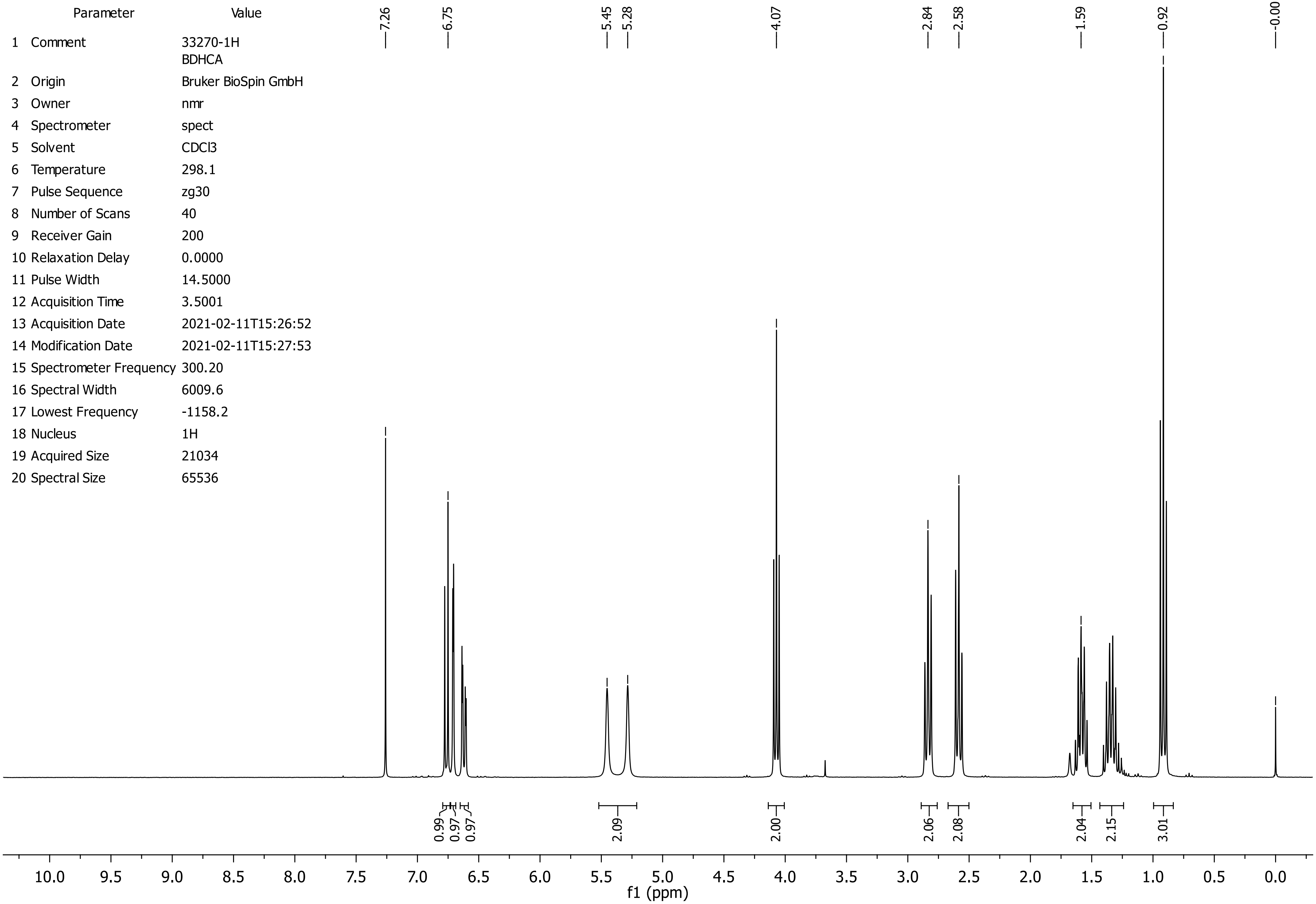

Supplement: Supplementary file 1 [file molecules-27-05024-s001.zip › 1HNMRfiles/1.pdf]
