# Supplementary material for: Lipase-Catalyzed Synthesis, Antioxidant Activity, Antimicrobial Properties and Molecular Docking Studies of Butyl Dihydrocaffeate
Source: Molecules. 2022 Aug 7;27(15):5024. doi: 10.3390/molecules27155024 (PMC9370587; doi:10.3390/molecules27155024)
Supplement: Supplementary file 1 [file molecules-27-05024-s001.zip › Zieniuk Supplementary materials.pdf]

molecules

Supplementary Material

Lipase-Catalyzed Synthesis, Antioxidant Activity, Antimicrobial Properties and Molecular Docking Studies of Butyl Dihydrocaffeate

Bartłomiej Zieniuk [1,*], Chimaobi James Ononamadu [2], Karina Jasińska [1,3], Katarzyna Wierzchowska [1,3] and Agata Fabiszewska [1]

[1] Department of Chemistry, Institute of Food Sciences, Warsaw University of Life Sciences—SGGW, 159c Nowoursynowska St., 02-776 Warsaw, Poland; karina_jasinska@sggw.edu.pl (K.J.), katarzyna_wierzchowska1@sggw.edu.pl (K.W.), agata_fabiszewska@sggw.edu.pl (A.F.)
[2] Department of Biochemistry and Forensic Science, Nigeria Police Academy, Wudil, Kano State, Nigeria; ononamaducj0016@gmail.com (C.J.O.)
[3] Department of Food Engineering and Process Management, Warsaw University of Life Sciences—SGGW, 159c Nowoursynowska St., 02-776 Warsaw, Poland;
* Correspondence: bartlomiej_zieniuk@sggw.edu.pl; Tel.: +48-22-59-37-621 (B.Z.)

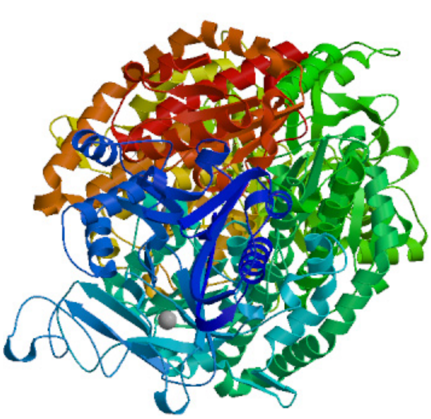

(**a**)

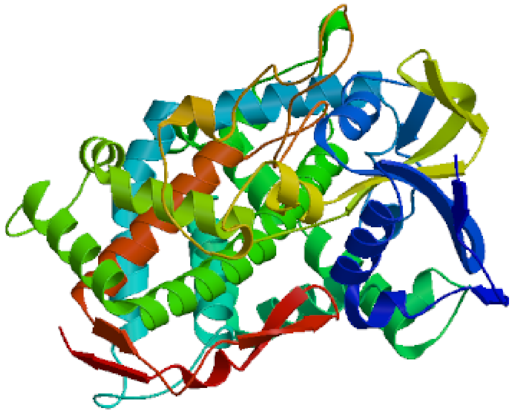

(**b**)

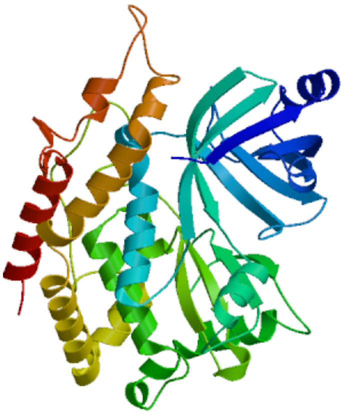

(**c**)

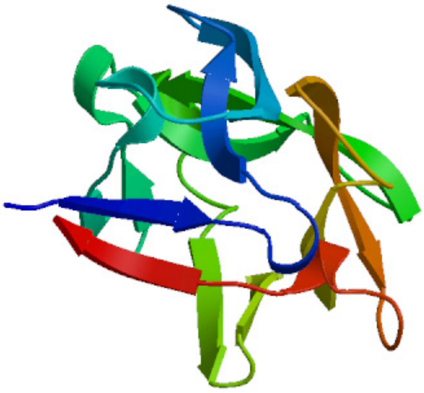

(**d**)

Figure S1. Structures of the modelled proteins (**a**) glutamine-fructose-6-phosphate transaminase, (**b**) 14-α sterol demethylase B, (**c**) invasin CotH3, (**d**) mucoricin.

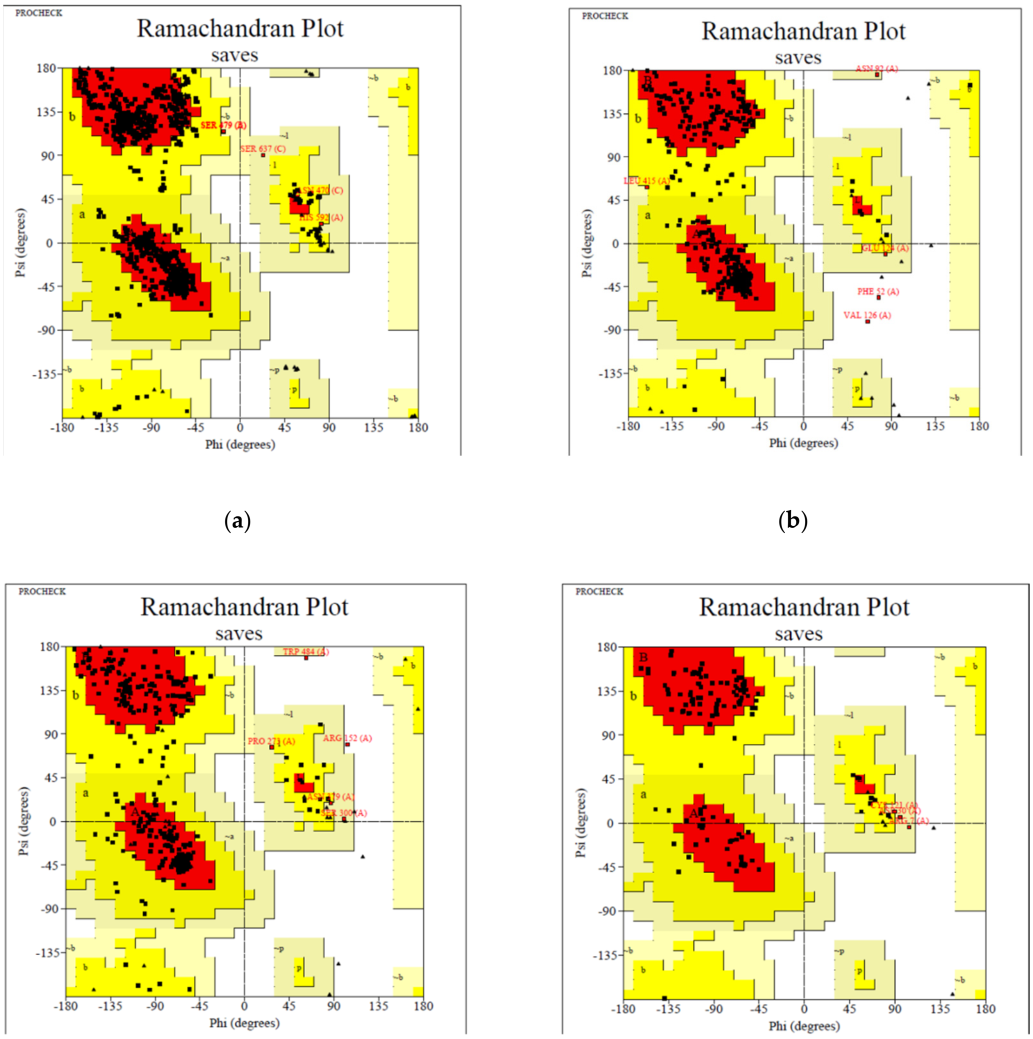

(**a**) (**b**)

(**c**) (**d**)

Figure S2. The Ramachandran's plots (PROCHECK) of the modelled proteins (**a**) glutamine-fructose-6-phosphate transaminase, (**b**) 14-α sterol demethylase B, (**c**) invasin CotH3, (**d**) mucoricin.

Table S1. The estimated target-template alignment / predicted model quality indices.

	**GFAT**	**14-α sterol demethylase B**	**Invasin CotH3**	**Mucoricin**
	SWISSPROT			
Template	6r4g.1.A	4uhl.1.A	5jd9.1.A	3pg0.1.A
Sequence Identity (%)	59.88	47.15	19.92	32.09
Coverage	0.98	0.86	0.91	0.97
Sequence Similarity	0.48	0.43	0.31	0.37
GMQE	0.83	0.67	0.59	0.68
QMEANDisCo Global	0.80 ± 0.05	0.70 ± 0.05	0.56 ± 0.05	0.68 ± 0.07
	ERRAT			
Overall Quality Factor	95.02	92.31	86.13	87.12
	PROCHECK			
Residues in Most Favoured Regions	91.70%	89.60%	84.30%	82.50%
Residues in Additional Allowed Regions	7.90%	9.20%	14.60%	15.10%
Residues in Generously Allowed Regions	0.40%	0.70%	0.60%	2.40%
Residues in Disallowed Regions	0.00%	0.50%	0.60%	0.00%

Abbreviations: GFAT - glutamine-fructose-6-phosphate transaminase, GMQE - Global Model Quality Estimate
