# Supplementary figures and images for: Lipase-Catalyzed Synthesis, Antioxidant Activity, Antimicrobial Properties and Molecular Docking Studies of Butyl Dihydrocaffeate
Source: Molecules. 2022 Aug 7;27(15):5024. doi: 10.3390/molecules27155024 (PMC9370587; doi:10.3390/molecules27155024)

| Parameter | Value     |
|-----------|-----------|
| Comment   | 42094-13C |
|           | B1        |

—115.363  
—115.316

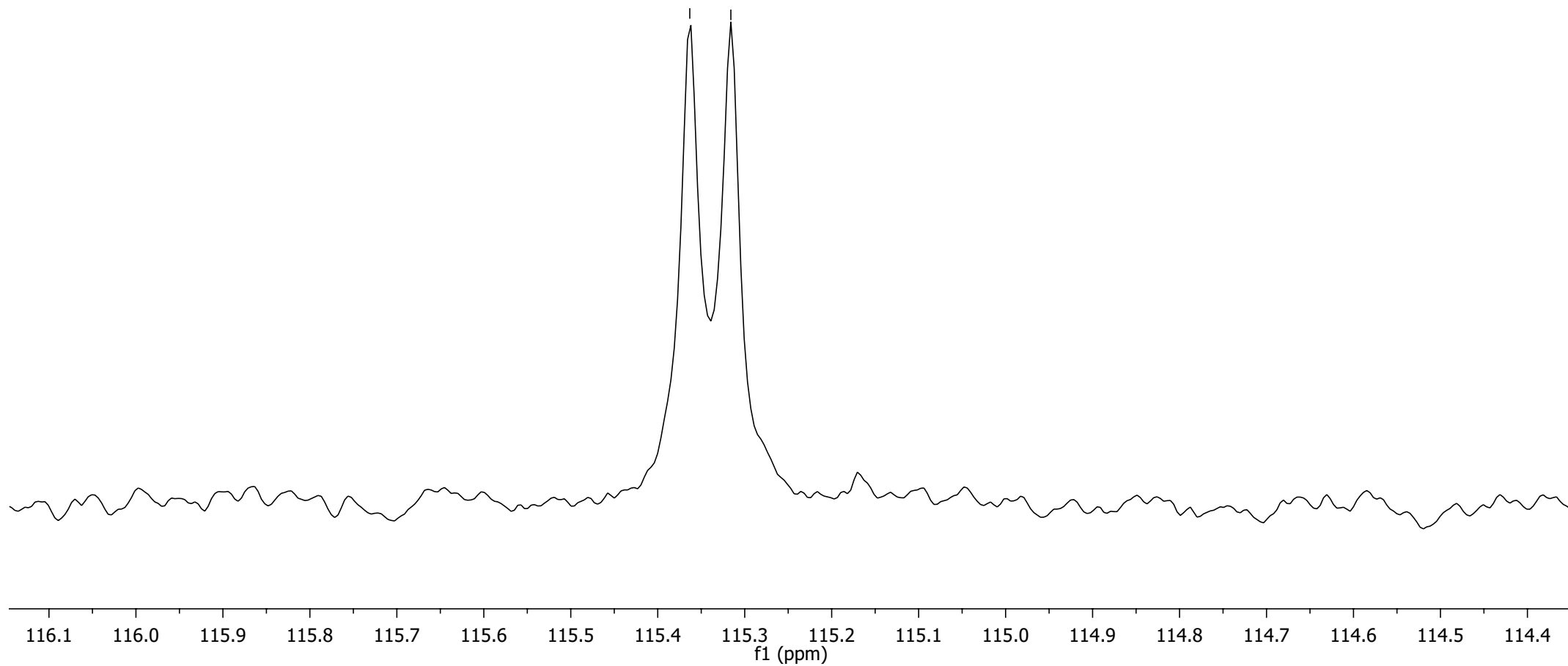

Supplement: Supplementary file 1 [file molecules-27-05024-s001.zip › 13CNMRfiles/2.pdf]

| Parameter | Value     |
|-----------|-----------|
| Comment   | 42094-13C |
|           | B1        |

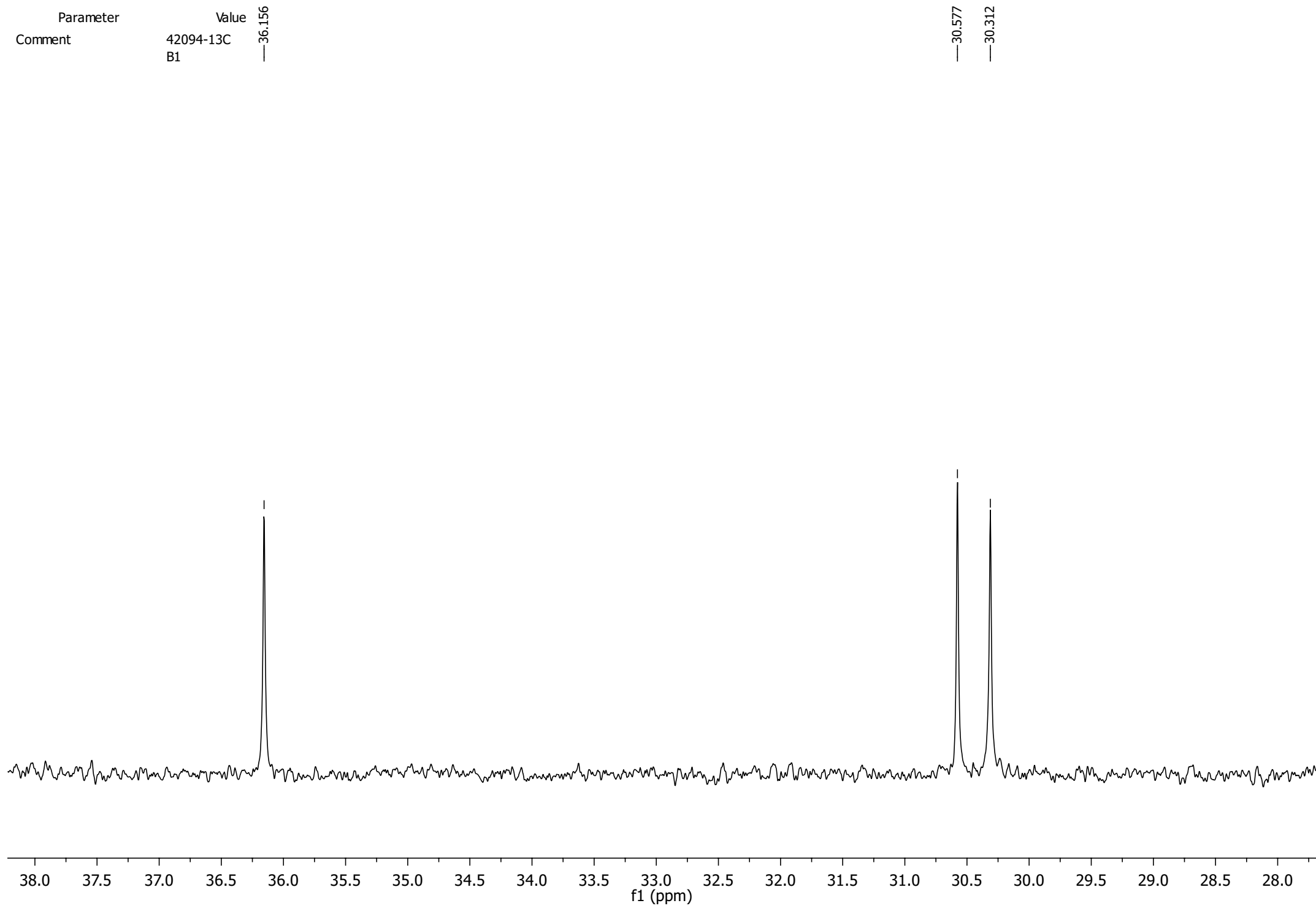

Supplement: Supplementary file 1 [file molecules-27-05024-s001.zip › 13CNMRfiles/3.pdf]

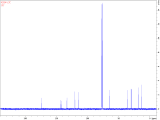

Supplement: Supplementary file 1 [file molecules-27-05024-s001.zip › 13CNMRfiles/rawdata/pdata/1/thumb.png]

6.78  
6.75  
6.71  
6.70

6.64  
6.63  
6.61  
6.60

5.45

5.28

| Parameter | Value             |
|-----------|-------------------|
| 1 Comment | 33270-1H<br>BDHCA |

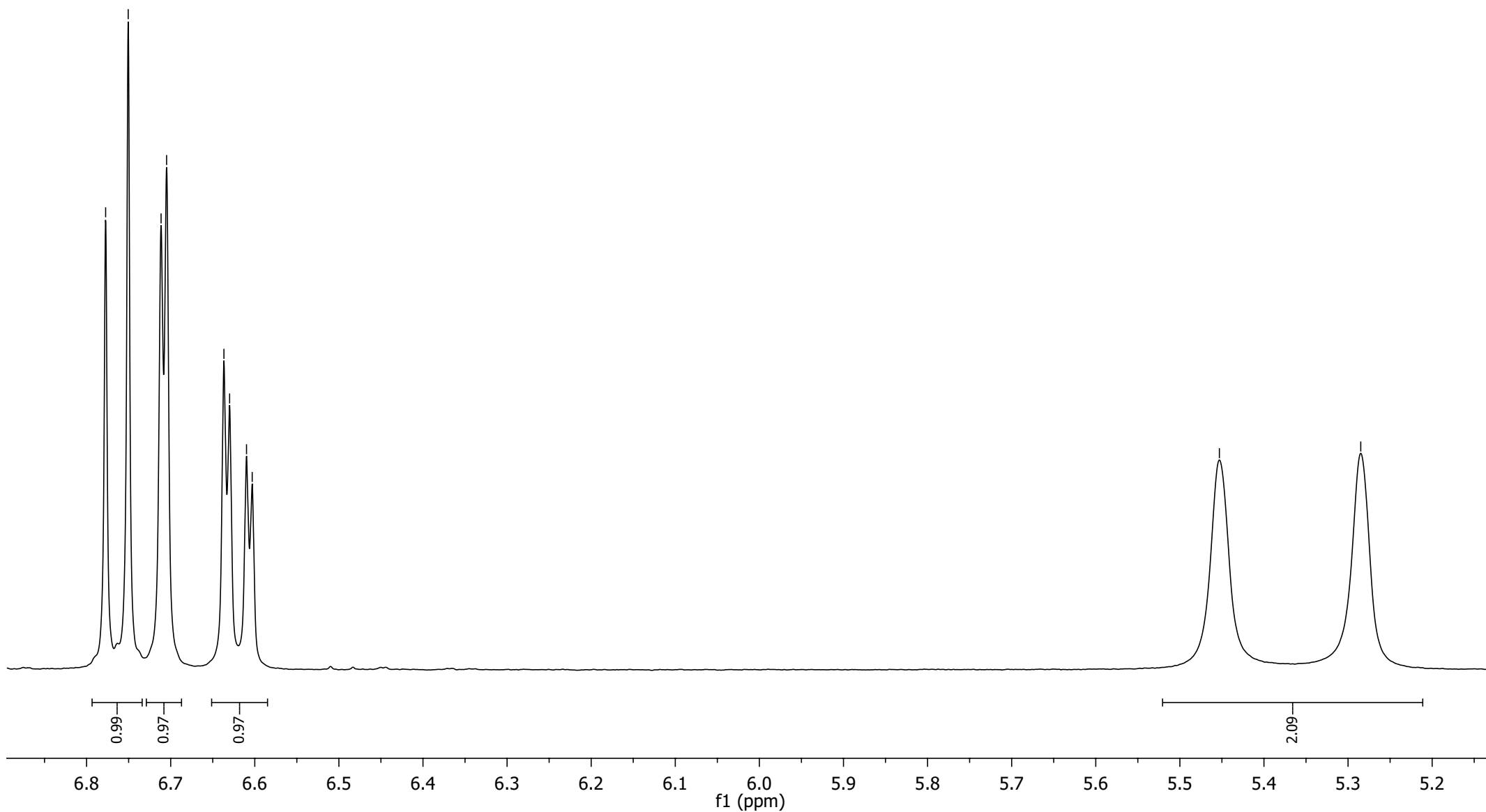

Supplement: Supplementary file 1 [file molecules-27-05024-s001.zip › 1HNMRfiles/2.pdf]

4.09  
4.07  
4.05

2.86  
2.84  
2.81

2.61  
2.58  
2.56

|   | Parameter | Value             |
|---|-----------|-------------------|
| 1 | Comment   | 33270-1H<br>BDHCA |

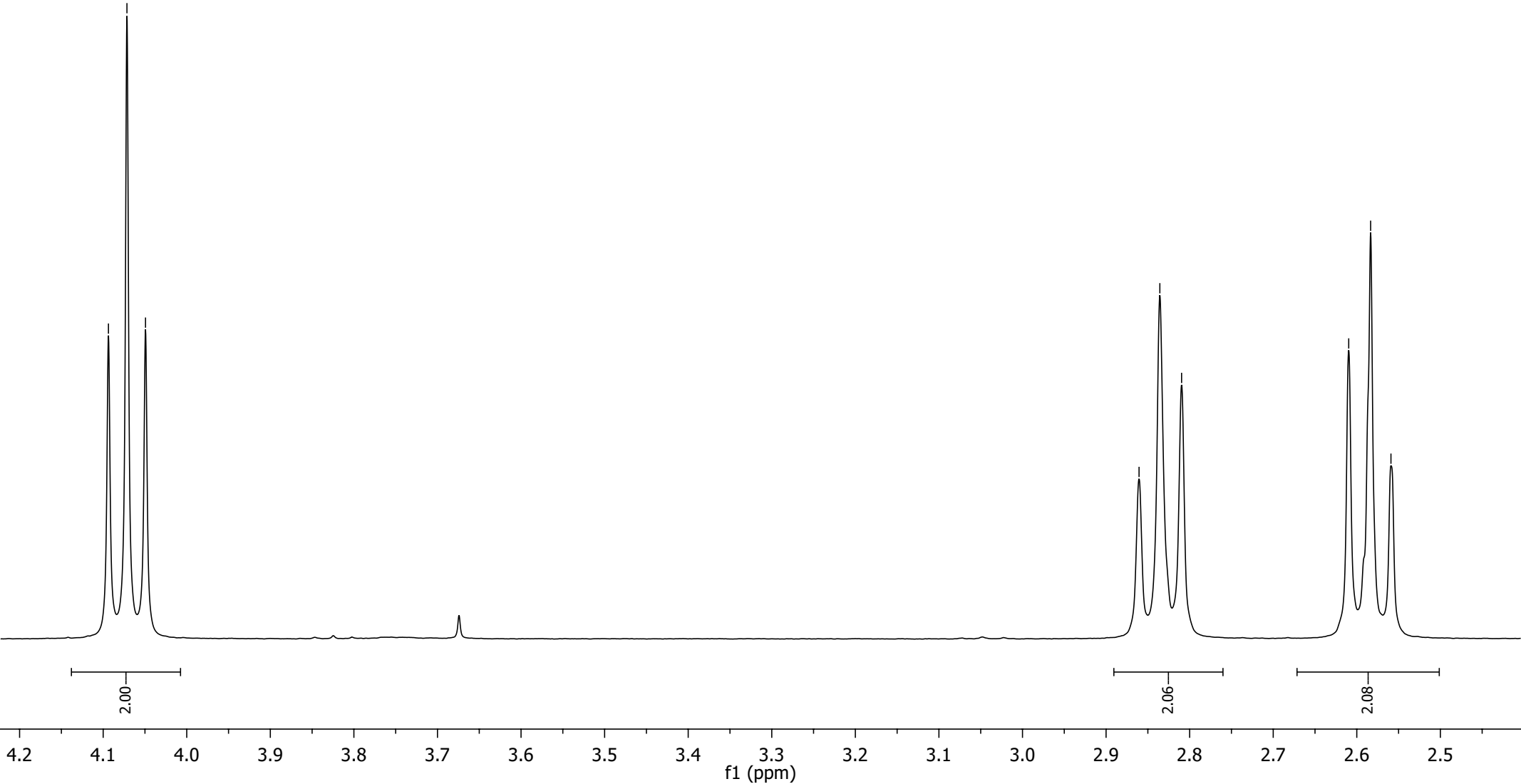

Supplement: Supplementary file 1 [file molecules-27-05024-s001.zip › 1HNMRfiles/3.pdf]

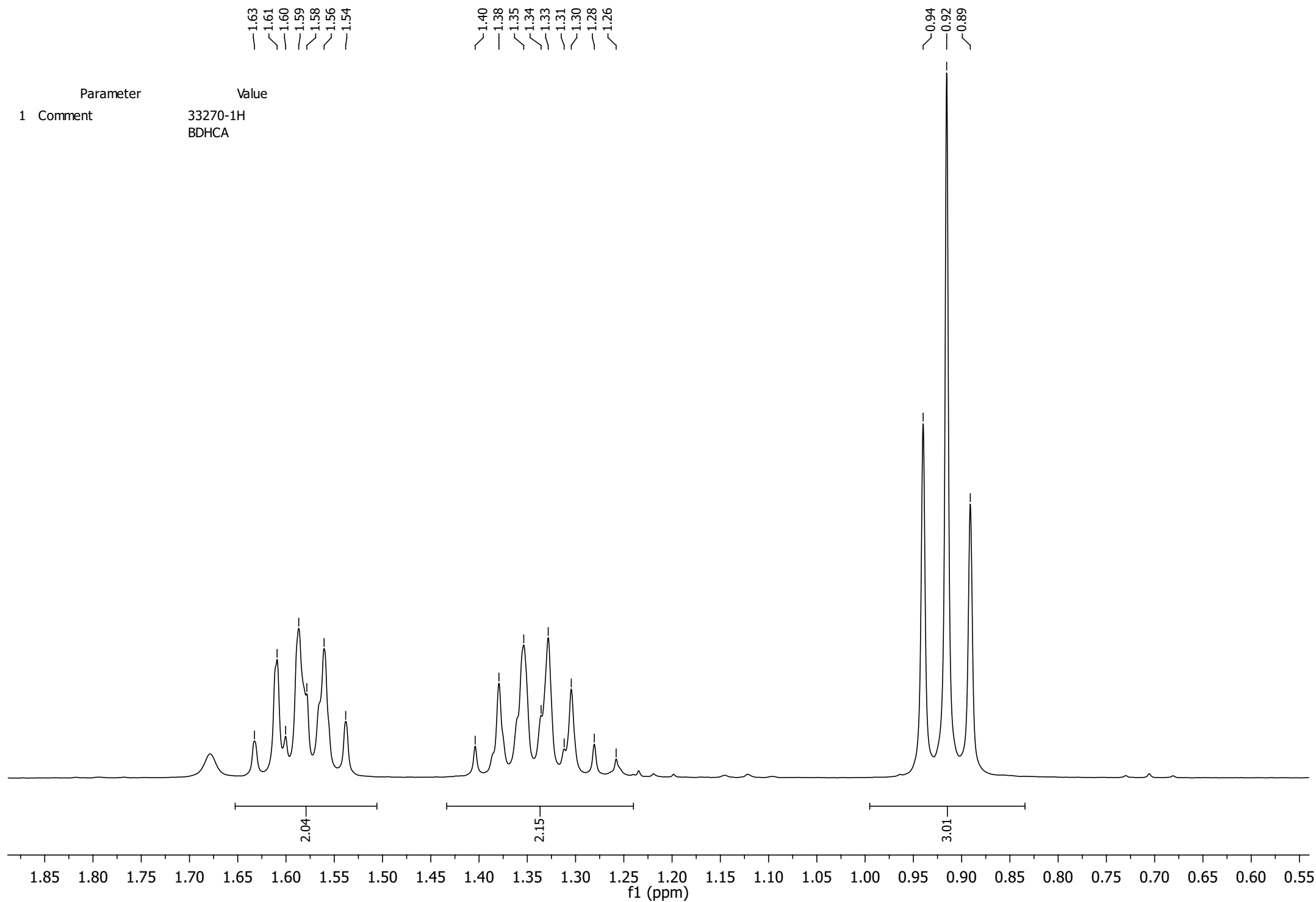

Supplement: Supplementary file 1 [file molecules-27-05024-s001.zip › 1HNMRfiles/4.pdf]

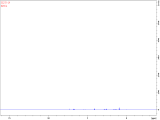

Supplement: Supplementary file 1 [file molecules-27-05024-s001.zip › 1HNMRfiles/rawdata/pdata/1/thumb.png]
